# Integrative Analysis of Telomere‐Related Genes Reveals Prognostic Signatures in Laryngeal Cancer

**DOI:** 10.1155/ijog/5301600

**Published:** 2026-04-08

**Authors:** Yesong Cheng, Yingjie Zhao, Lin He, Dingqiang Huang, Feipeng Zhao, Xiangyang Shi, Wensong Tang, Yi Liu, Wujun Zou, Xiaolong Tang, Yi He

**Affiliations:** ^1^ Otolaryngology Head and Neck Surgery, Chengdu Second People’s Hospital, Chengdu, Sichuan, 610011, China, cd2120.com; ^2^ Department of Pathology, Sichuan Provincial Maternity and Child Health Care Hospital, Chengdu, Sichuan, 610045, China, scu.edu.cn; ^3^ Department of Pathology, Chengdu Second People’s Hospital, Chengdu, Sichuan, 610011, China, cd2120.com; ^4^ The Second Department of Gastrointestinal Surgery, Affiliated Hospital of North Sichuan Medical College, Nanchong, Sichuan, 638700, China, hospital-nsmc.com.cn

**Keywords:** bioinformatics, immune microenvironment, laryngeal cancer, prognostic biomarkers, telomeres

## Abstract

**Background:**

Laryngeal cancer (LC) is a common malignant tumor. Telomere‐related genes (T‐RGs) play critical roles in cellular senescence and carcinogenesis, but their prognostic relevance in LC remains to be fully elucidated. Therefore, exploring the prognostic genes related to telomeres in LC is important.

**Methods:**

Public retrospective datasets TCGA‐HNSC and T‐RGs were used to identify candidate genes by intersecting differentially expressed genes with T‐RGs. Key analytical approaches, including machine learning algorithms and univariate Cox regression, were applied to identify prognostic genes and construct a prognostic model. A nomogram was developed to assess the prognostic value for LC based on overall survival. Disease samples were classified into high‐risk and low‐risk groups, and subsequent analyses included immune infiltration, immune checkpoint expression, and related evaluations. Experimental validation of prognostic genes was performed through RT‐qPCR.

**Results:**

A total of 314 candidate genes were obtained from 8961 differentially expressed genes. Four key prognostic genes (CHTF18, FANCG, NR5A1, and XRCC3) were identified. The constructed risk score model retained consistent predictive accuracy in both the training and validation datasets, with AUCs ranging from approximately 0.61 to 0.9. Enriched activated immune cells were detected in the low‐risk group through immune microenvironment analysis, whereas immune suppression–related features were identified in the high‐risk group, accompanied by a reduced tumor mutational burden that was detected. Finally, preliminary validation using RT‐qPCR in a limited cohort of clinical samples indicated that the expression trends of three prognostic genes were elevated in LC tissues, showing concordance with the bioinformatic findings.

**Conclusions:**

This study identified four key prognostic T‐RGs (CHTF18, FANCG, NR5A1, and XRCC3) and constructed a corresponding prognostic model for LC. Our analyses further suggest a potential link between telomere maintenance mechanisms and the tumor immune microenvironment, which may influence patient outcomes.

## 1. Introduction

Laryngeal cancer (LC), predominantly occurring as laryngeal squamous cell carcinoma, is a common malignancy accounting for 20%–30% of all head and neck cancers, imposing a significant public health burden globally [1]. Clinically, LC presents with hoarseness, dysphagia, dyspnea, and hemoptysis. The onset of the disease is associated with multiple factors, including smoking, alcohol consumption, and HPV infection [2]. Treatment primarily involves surgery, radiotherapy, and chemotherapy. Early diagnosis and standardized treatment can improve prognosis [3]. In recent years, novel therapeutic approaches have emerged, including the use of nanomaterials [4, 5], immunotherapy [6–8], and microbiome‐based interventions [9], which show promise in improving the treatment outcomes for LC. Despite advances in diagnostic and therapeutic techniques, most patients are still diagnosed at advanced stages due to nonspecific early symptoms, limiting effective treatment and prognosis. Thus, identifying novel biomarkers and therapeutic targets remains imperative for improving LC management [10].

Telomeres, evolutionarily conserved protein–DNA complexes at chromosome ends, maintain chromosomal stability by preventing genetic information loss during cell division [11]. Progressive telomere shortening occurs naturally with each cellular replication in the absence of telomerase, eventually triggering cellular senescence or apoptosis [12]. Notably, heightened telomerase activity, observed in 85%–95% of human cancers, facilitates indefinite tumor cell proliferation and survival, playing a crucial role in tumor progression and therapeutic resistance [13]. Prior studies have demonstrated that shorter telomeres correlate with poorer differentiation and prognosis in laryngeal squamous cell carcinoma patients [14]. Additionally, several telomere‐related genes (T‐RGs), such as oligosaccharide‐binding fold containing 1 (OBFC1), have been associated with LC tumorigenesis [15]. This indicates that telomeres and their related genes play a key role in various types of cancer, but their specific functions in the pathogenesis and prognosis of LC remain unclear. Therefore, it is crucial to conduct in‐depth research into their molecular mechanisms and clinical significance.

This study aims to systematically identify and characterize key T‐RGs in LC, with the goal of exploring their potential associations with tumorigenesis, progression, and clinical outcomes in LC. We first retrieved LC transcriptomic data and screened for T‐RGs, utilizing univariate Cox regression and LASSO regression analyses to identify core genes closely associated with prognosis, upon which a prognostic risk model was constructed. Subsequently, we conducted comprehensive bioinformatic analyses, including molecular regulatory network construction centered on these pivotal genes to thoroughly explore their functional mechanisms in LC progression. The findings of this research are expected to provide new theoretical insights and identify key T‐RGs in LC, contributing to a better understanding of their potential roles in LC.

## 2. Materials and Methods

### 2.1. Data Collection

We filtered the TCGA‐HNSC clinical data by selecting cases labeled as “Larynx, NOS” in the tissue_or_organ_of_origin.diagnoses field to isolate LC samples. Initially, there were 604 samples, and after filtering, 139 samples remained, consisting of 115 LC tissue samples and 12 control samples. The TCGA‐HNSC dataset was obtained from Xena (https://xenabrowser.net/), and we also downloaded prognostic information, somatic mutation data, tumor mutation burden (TMB), and clinical data. During machine learning analysis, the LC cancer tissue sample data in the training set were randomly divided into a 7:3 ratio, 70% samples were defined as training set for differential analysis and construction of prognostic models, and 30% of the samples were defined as the validation set to confirm the reliability of the prognostic model. The T‐RGs were sourced from TelNet (https://www.cancertelsys.org/telnet/) using the criteria of TelNet score ≥ 1 (v2024.11.17) [16] (Supporting Table 1).

### 2.2. Identification of Differentially Expressed Genes

Differential expression analysis was performed using the “DESeq2” package (v1.40.2) [17], comparing tumor samples with matched normal tissues from the TCGA‐HNSC cohort. Genes were considered significantly differentially expressed when *p*.adjust (adj.*p*) was < 0.05 and |log2 FC| > 1. The volcano plot was employed to visually represent the DEGs by “ggplot2” (v3.5.1) [18] package, and the top 10 genes sorted by log2FC were labeled. Concurrently, the “ComplexHeatmap” package (v2.16.0) [19] was applied to visualize the top 20 DEGs in the form of a heatmap.

### 2.3. Functional Annotation and Network Analysis of Candidate Genes

The overlap between DEGs and T‐RGs was identified using the “ggvenn” package (v0.1.10) [20], and the intersecting genes were defined as candidate genes. Functional enrichment analysis of these genes was conducted with “clusterProfiler” (v4.15.0.003) [21], where significantly enriched GO terms and KEGG pathways (*p* < 0.05) were retained. Protein–protein interactions were retrieved from the STRING database (https://string-db.org/, organism = *Homo sapiens*, interaction score ≥ 0.9) and visualized in Cytoscape (v3.9.1) [22]. Core genes were extracted by applying a degree cutoff of ≥ 2 in the PPI network. Chromosomal distribution of candidate genes was illustrated using the “Circos” package [23].

### 2.4. Acquisition of Prognostic Genes

Seventy percent of TCGA‐HNSC samples were randomly assigned to the training set, while the remaining 30% were used as the validation set. In the training cohort, univariate Cox regression and LASSO analyses were performed to identify prognostic genes. To screen for genes associated with overall survival (OS), univariate Cox analysis was conducted using the “survival” package (v3.5‐3) [24] (HR ≠  1, *p* < 0.05), and the results were visualized with the “forestplot” package (*p* < 0.05). A proportional hazards (PH) assumption test (*p* > 0.05) was then applied to the univariate Cox results via the “ggcoxzph” function in the “survival” package. Subsequently, LASSO regression was carried out with the “glmnet” package (v4.1‐8) [25] using 10‐fold cross‐validation to select the optimal *λ* value, and the genes corresponding to this model were retained as prognostic genes. Finally, the Cell‐PLoc 2.0 tool was employed to predict the subcellular localization of the proteins encoded by these prognostic genes.

### 2.5. Prognostic Model Construction and Validation

Risk scores were calculated for TCGA‐HNSC samples with available survival information. The calculation formula was as follows:

(1) Risk score=∑i=1ncoefi×Ai.

Here, *coef* represents the coefficient and *A*_*i*_ denotes the gene expression. In the training set, patients were divided into high‐risk group (HRG) and low‐risk group (LRG) according to the median risk score. Survival status and risk distribution were visualized with the “survminer” package (v0.4.9) [26], and group differences were assessed by Kaplan–Meier analysis with the log‐rank test using the “survival” package (v3.7.0) [26]. The predictive performance of the model was further evaluated using time‐dependent ROC curves at 1, 3, and 5 years generated with the “timeROC” package (v0.4) [27]. The same analyses were subsequently applied to the validation set.

### 2.6. Correlation Analysis of Risk Score With Clinical Features

Samples from TCGA‐HNSC with available clinical data and risk scores were included in the analysis. Associations between risk score and clinical subgroups (age, gender, race, stage, and grade) were evaluated using the log‐rank test (*p* < 0.05). In addition, Spearman correlation analysis was conducted to further assess relationships between clinical characteristics and risk score (|cor| > 0.3, *p* < 0.05).

### 2.7. Gene Set Enrichment Analysis (GSEA) and Gene Set Variation Analysis (GSVA)

Using c2.cp.kegg.v7.4.symbols.gmt gene set in MSigDB as reference, the “GSVA” package was implemented to gauge ssGSEA scores of the KEGG pathway between HRG and LRG. And the “limma” (v3.56.2) [28] package was used to identify the pathway variation in two groups (|*t*| > 2, *p* < 0.05). The difference analysis of HRG and LRG was performed by the “DEseq2” package, the corresponding log2FC was calculated, and the log2FC was sorted in descending order and analyzed with the “clusterProfiler” package (v4.15.0.003) [29] (*p* < 0.05). The top 10 pathways sorted in descending order of the absolute value of NES were presented (FDR < 0.25, *p* < 0.05, |NES| > 1).

### 2.8. Analysis of Immune Microenvironment and Immune Characteristics

The CIBERSORT algorithm was applied to the TCGA‐HNSC training dataset to estimate the distribution of immune cells, excluding samples with *p* > 0.05. Differences in immune cell proportions between the HRG and LRG were assessed using the Wilcoxon test, and significantly altered immune cells (*p* < 0.05) were identified. Correlations between prognostic genes and immune cell fractions were analyzed with the “psych” package (v2.4.3) [30] (|cor| > 0.3, *p* < 0.05). In addition, expression differences of immune checkpoint genes [31] between the two risk groups were evaluated by the Wilcoxon test, and further correlation analyses were performed to explore associations among prognostic genes, risk scores, and checkpoint expression (|cor| > 0.3, *p* < 0.05). The TIDE score was calculated using the online tool TIDE (https://tide.dfci.harvard.edu/), and differences in TIDE scores between the HRG and LRG were compared using the Wilcoxon test.

### 2.9. Mutation Landscape Analysis

The cBioportal database (https://www.cbioportal.org/) was used to obtain sample data. The TMB of the HRG and LRG was investigated by “maftools” (v2.16.0) [32] (*p* < 0.05). The “maftools” package was used to compare sample data and somatic mutation database to check the two risk groups of mutations that exist in the sample. The “plotmafSummary” function was used to display the mutation situations of the HRG and LRG.

### 2.10. Validation by RT‐qPCR

The expression of key genes was validated using RT‐qPCR in 5 LC and 5 matched control tissue samples collected from Chengdu Second People’s Hospital. The patients were histopathologically diagnosed with laryngeal squamous cell carcinoma. Patient information is provided in Supporting Table 2. Ethical approval was obtained from the Medical Ethics Committee of Chengdu Second People’s Hospital (Approval No. [KY]PJ2024144), and written informed consent was provided by all participants. Total RNA was extracted with TRIzol reagent (Vazyme, Nanjing, China) and reverse‐transcribed into cDNA using the HP All‐in‐One qRT Master Mix II, RT203‐Ver.1 (Younggenbio, Kunming, China), following the manufacturer’s protocols. qPCR was conducted with 2× Universal Blue SYBR Green Master Mix (Servicebio, Wuhan, China), and results were analyzed in GraphPad Prism 10. GAPDH served as the internal reference, and relative gene expression was calculated using the 2^−ΔΔCt^ method. For each sample, three technical replicates were performed in qPCR, and the average Ct value was used. ΔCt was calculated as the difference between the Ct values of the target gene and the internal reference gene, and ΔΔCt was calculated as the difference between the ΔCt values of the experimental and control groups. Primer sequences are listed in Table 1.

**TABLE 1:** List of primers.

Primers	Sequence
CHTF18 F	CACGCCCCCAGGATCAAA
CHTF18 R	CAG​CTT​CTG​AGA​CTC​CGT​GG
FANCG F	AGC​CTC​ACC​CCT​TCA​TTG​TG
FANCG R	TCA​AGG​CAT​CTT​GGG​CTC​TG
NR5A1 F	CCC​ACT​AGG​TGA​ACA​GCA​GG
NR5A1 R	GCC​ACA​GAG​AGG​GGA​TCA​AC
XRCC3 F	GGA​AGA​GGA​GTG​CGG​AAC​C
XRCC3 R	CTG​TCA​CTC​TCT​GGG​GCT​TG
GAPDH F	ATG​GGC​AGC​CGT​TAG​GAA​AG
GAPDH R	AGG​AAA​AGC​ATC​ACC​CGG​AG

### 2.11. Statistical Analysis

All analyses were executed utilizing R programming language (v4.3.2). The Wilcoxon and Kruskal–Wallis tests were harnessed to contrast differences, employing a statistical significance threshold of *p* < 0.05 or adj.*p* < 0.05.

## 3. Results

### 3.1. Enrichment Analysis of Candidate Genes

Based on a TelNet score ≥ 1, we identified 2086 T‐RGs from TelNet. The differential expression analysis was performed between the LC and the control samples in training set of TCGA‐HNSC. The clinical data characteristics of the patients are provided in Supporting Table 3. Altogether 9275 DEGs were identified including 6887 up‐ and 2388 downregulated genes in LC (Figures 1(a) and 1(b)) (Supporting Table 4). Subsequently, the intersection of the 9275 DEGs and 2086 T‐RGs was taken, and 314 candidate genes were obtained (Figure 1(c)). GO and KEGG enrichment analysis revealed 414 significantly enriched terms, including 277 biological process (BP) terms such as DNA recombination, 85 cellular component (CC) terms such as nuclear chromosome, and 39 molecular function (MF) terms such as catalytic activity, and 13 KEGG pathway such as DNA replication (Figure 1(d)) (Supporting Table 5). The chromosome location of candidate genes was also visualized (Figure 1(e)). Then, the PPI results showed that there were 256 pairs of interactions among 115 candidate genes (Figure 1(f)). Since the number of variables in the machine learning model should be less than the number of samples, we took degree ≥ 2 in the PPI network as the screening condition. Altogether 55 core genes that had more interaction were obtained and were further investigated (Figure 1(g)).

FIGURE 1Differential gene analysis. (a) Volcano plot and (b) heatmap illustrate the DEGs between LC samples and controls in the training set; the significance threshold for the volcano plot is |log2FC| > 1 and adj.*p* < 0.05. (c) Intersection of DEGs with T‐RGs identified the candidate genes, which were subjected to (d) GO and KEGG enrichment analyses. (e) Chromosomal distribution of candidate genes is shown. (f) A PPI network of the candidate genes was constructed using the STRING database, and (g) core genes were further identified with a threshold of degree ≥ 2 to generate the refined PPI network.

**Figure:**
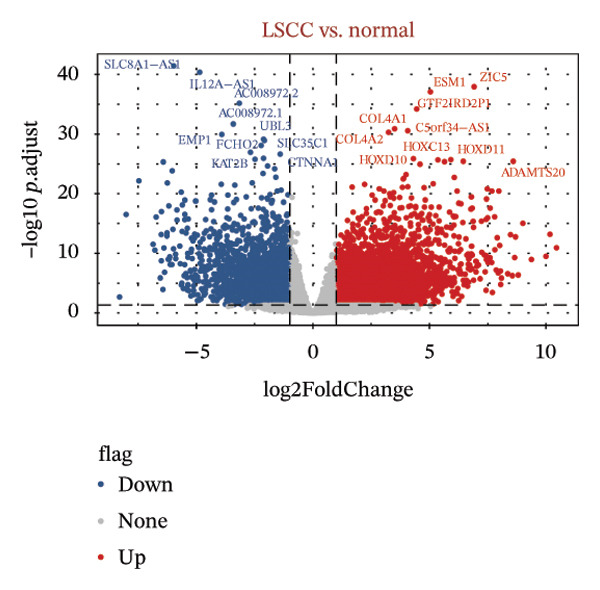
(a)

**Figure:**
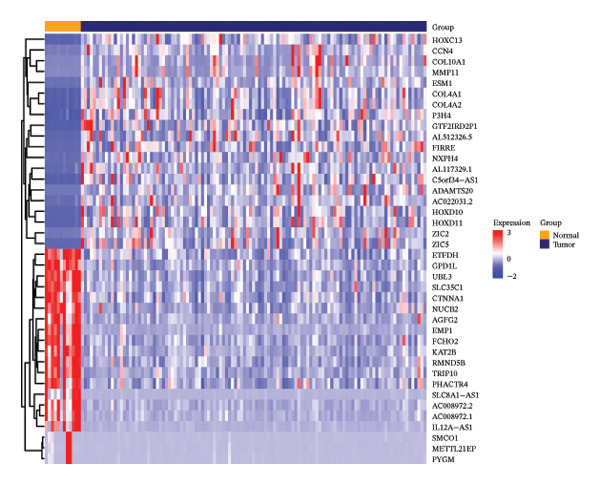
(b)

**Figure:**
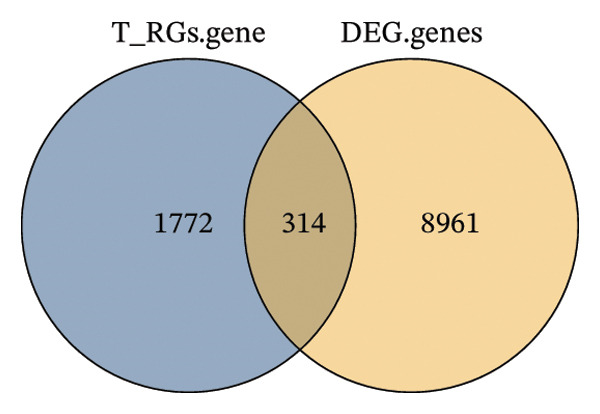
(c)

**Figure:**
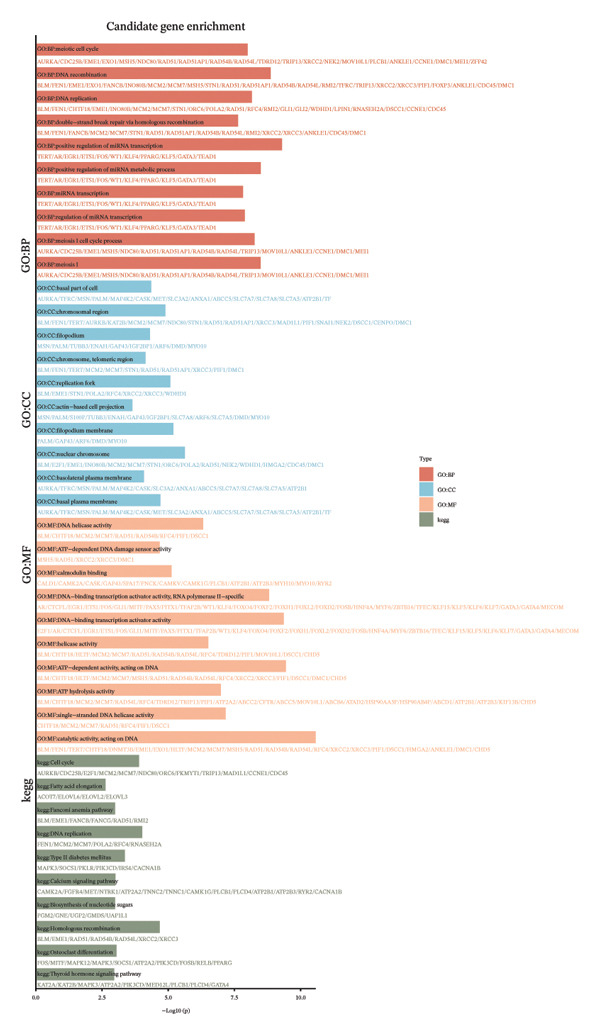
(d)

**Figure:**
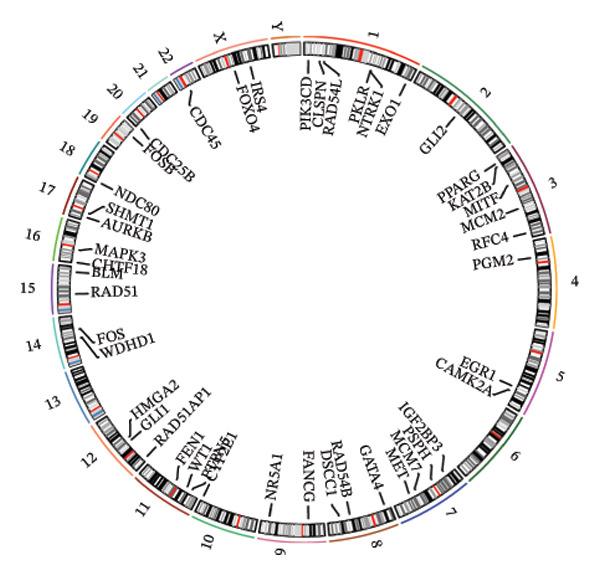
(e)

**Figure:**
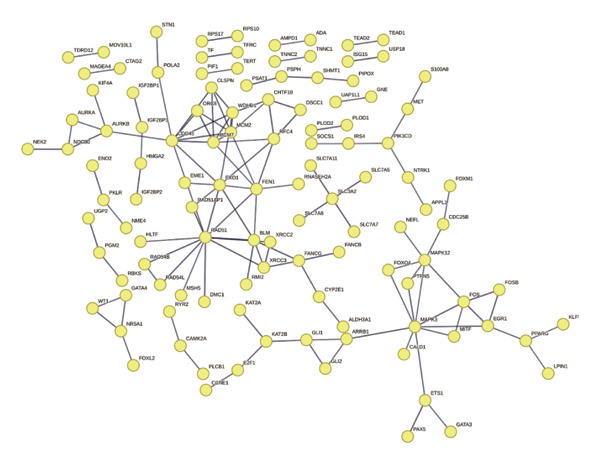
(f)

**Figure:**
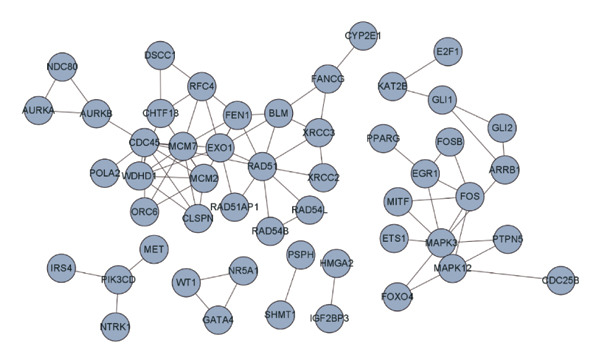
(g)

### 3.2. Four Prognostic Genes Were Identified

Univariate Cox regression (*p* < 0.05, HR ≠ 1) and PH assumption test (*p* > 0.05) of 55 core genes yielded 4 genes (Figures 2(a) and 2(b)). Then, LASSO regression analysis was conducted on 4 prognostic‐related genes. When lambda.min was 0.0282, 4 prognostic genes were finally screened (Figure 2(c)). Chromosome transmission fidelity factor 18 (CHTF18) and nuclear receptor subfamily 5 group A member 1 (NR5A1) were primarily localized in the cytoplasm, while Fanconi anemia complementation group G (FANCG) and X‐ray repair cross‐complementing 3 (XRCC3) were mainly found in the nucleus (Figure 2(d)).

FIGURE 2Identification of prognostic genes. (a) Univariate Cox analysis of 55 core genes identified four prognostic genes significantly associated with overall survival. (b) PH assumption testing using Schoenfeld residual plots confirmed that all four genes met the PH assumption. (c) LASSO regression was applied to further screen prognostic genes. (d) Subcellular localization of the four prognostic genes is shown.

**Figure:**
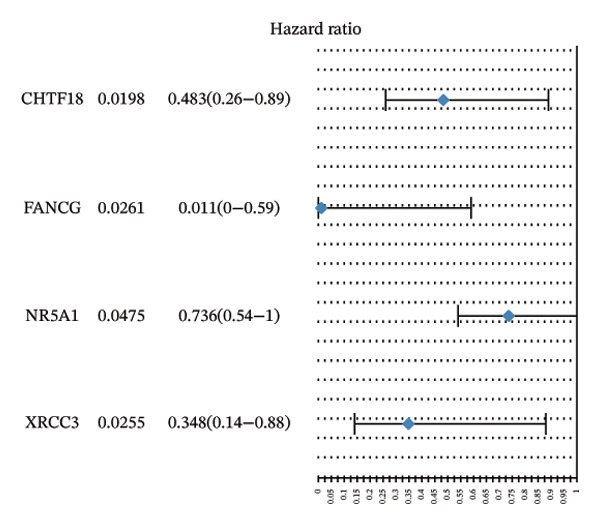
(a)

**Figure:**
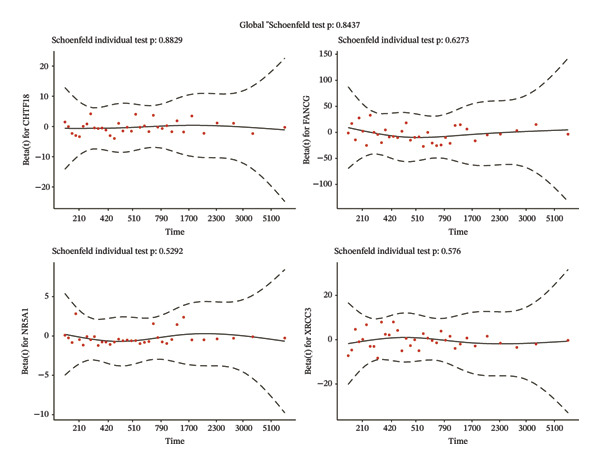
(b)

**Figure:**
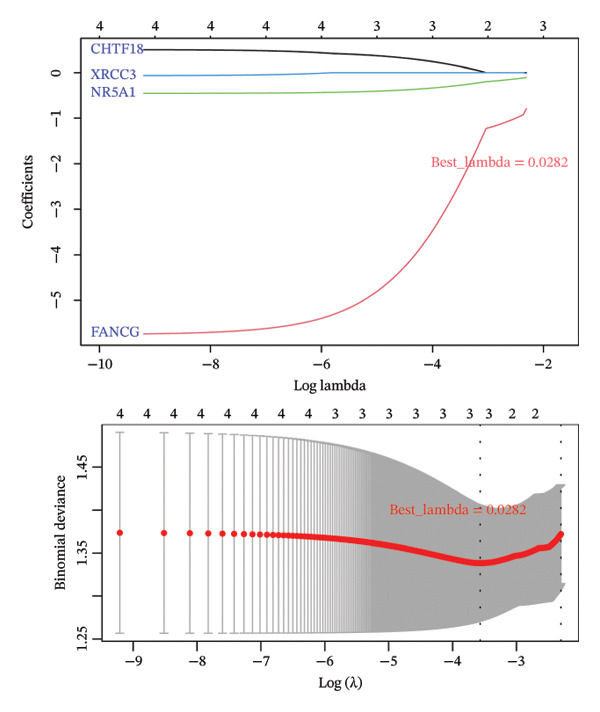
(c)

**Figure:**
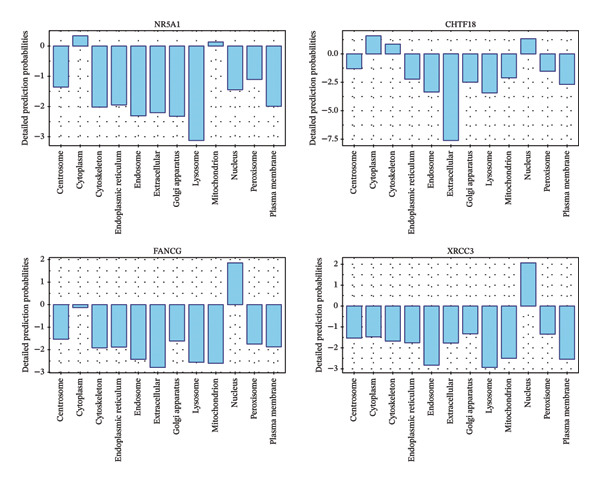
(d)

### 3.3. Prognostic Model Demonstrated a Good Prognostic Outcome on LC

According to above results, we get the formula of risk score: risk score = (expression of CHTF18 × (−0.04980998)) + (expression of FANCG × (−3.12301008)) + (expression of NR5A1 × (−0.29311927)) + (expression of XRCC3 × (−0.55725687)). Based on the median risk score (−1.71), samples of 70% TCGA‐HNSC were divided into HRG (40) and LRG (41). In the same manner, 34 LCs of 30% TCGA‐HNSC patients were also classified as HRG (17) and LRG (17) according to the median risk score (−2.28). In both 70% and 30% samples, the risk score distribution as plots showed that as the risk score rose, fatalities increased (Figures 3(a) and 3(d)). In both 70% (*p* = 0.011) and 30% (*p* = 0.015), the KM curves for both datasets showed that the survival was notably worse in HRG compared with LRG (Figures 3(b) and 3(e)). The AUCs of the prognostic model in ROC analysis at 1, 3, and 5 years were all greater than 0.6 (Figures 3(c) and 3(f)), which demonstrated the ability to forecast the survival of LC patients.

FIGURE 3Evaluation of the prognostic model in training and validation cohorts. (a, d) Distribution of risk scores and survival status in the training and validation sets. (b, e) Kaplan–Meier survival curves of high‐ and low‐risk groups. (c, f) Time‐dependent ROC curves at 1, 3, and 5 years, with AUCs of 0.61/0.77/0.67 in the training set and 0.89/0.77/0.68 in the validation set.

**Figure:**
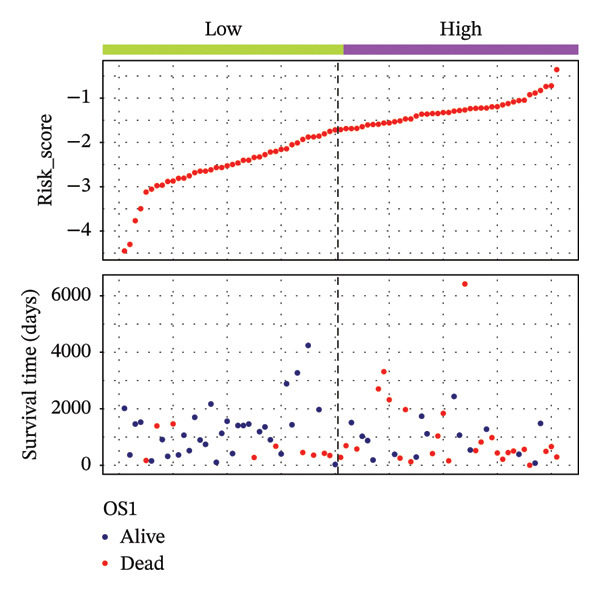
(a)

**Figure:**
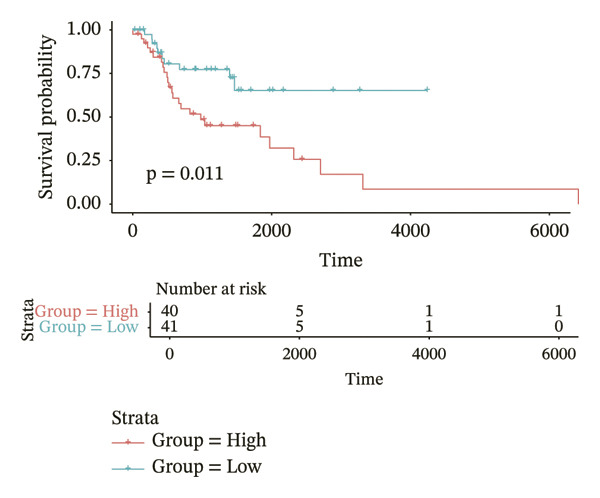
(b)

**Figure:**
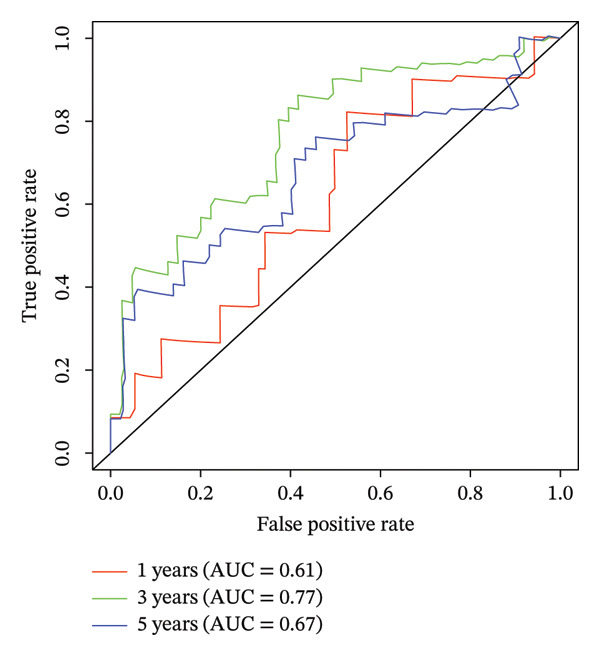
(c)

**Figure:**
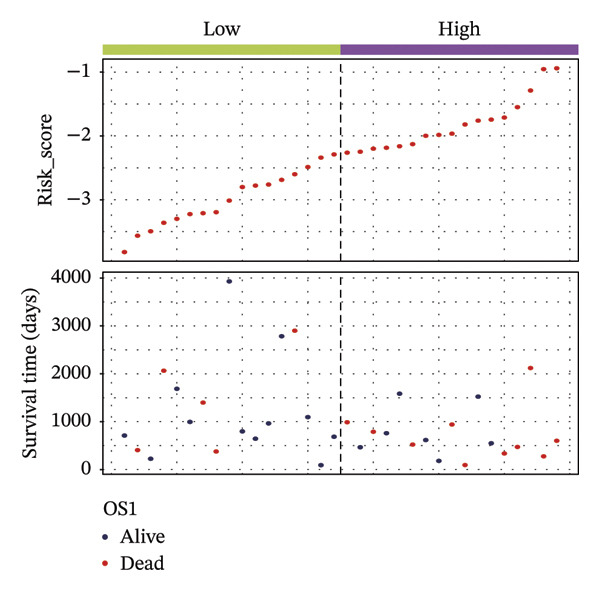
(d)

**Figure:**
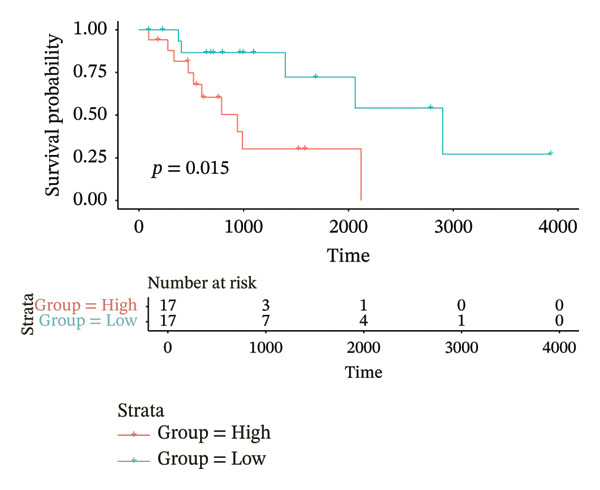
(e)

**Figure:**
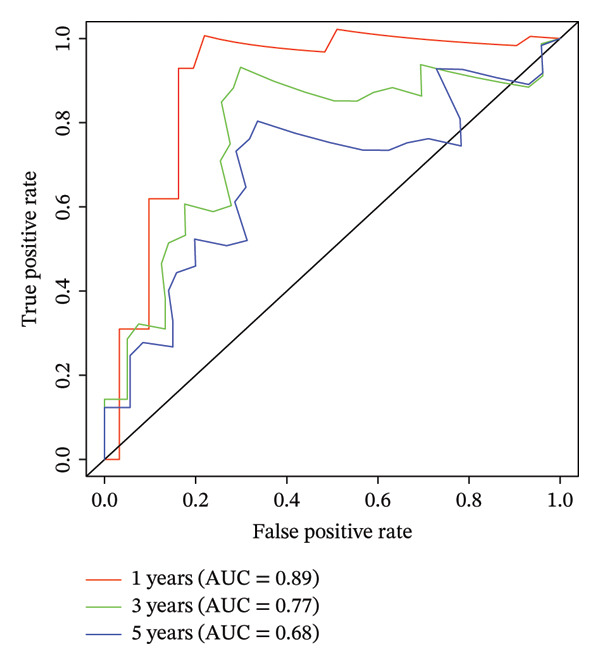
(f)

### 3.4. The Distribution of Risk Scores Across Clinical Characteristics

In different clinical characteristic subgroups, higher risk score linked to lower survival and a greater impact on survival in male patients (*p* = 0.0069), younger patients (aged < 60 years, *p* = 0.00025), patients with a history of alcohol consumption (*p* = 0.0016), nonsmoking patients (*p* = 0.011), patients with M0 stage (*p* = 0.0022), patients with Stage III (*p* = 0.015) and IV (*p* = 0.018), and patients with T3 (*p* = 0.0083) and T4 (*p* = 0.039) stages (Figures 4(a), 4(b), 4(c), 4(d), 4(e), 4(f), 4(g), 4(h), and 4(i)).

FIGURE 4Distribution of risk scores across different clinical subgroups. (a) KM survival curves of HRG and LRG in patients aged < 60 years. (b) KM survival curves of patients with alcohol consumption. (c) KM survival curves of patients without distant metastasis (M0). (d) KM survival curves of male patients. (e) KM survival curves of nonsmokers. (f) KM survival curves of patients with Stage III disease. (g) KM survival curves of patients with Stage IV disease. (h) KM survival curves of patients with T1–T3 stage. (i) KM survival curves of patients with T1–T4 stage.

**Figure:**
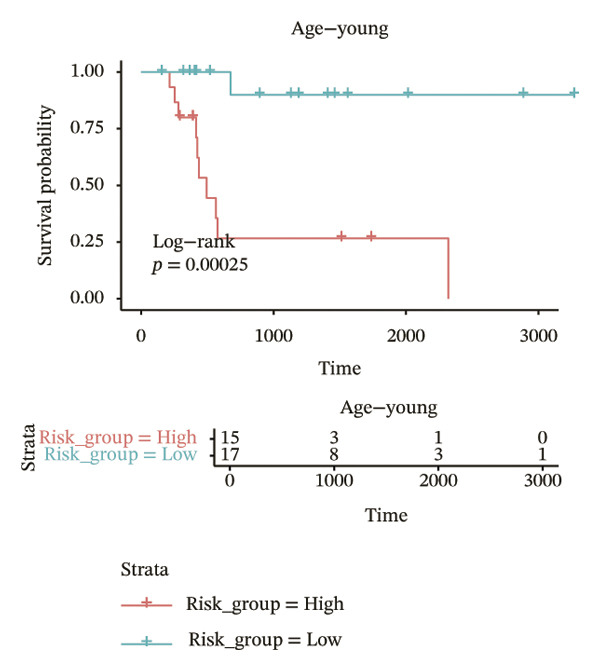
(a)

**Figure:**
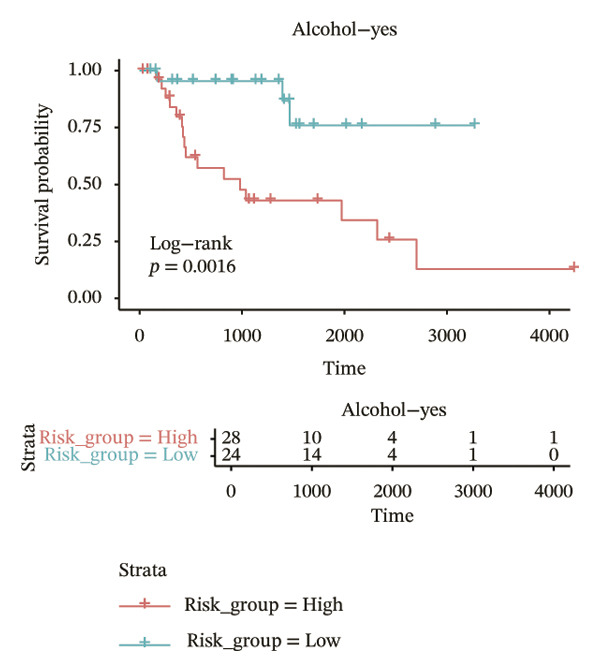
(b)

**Figure:**
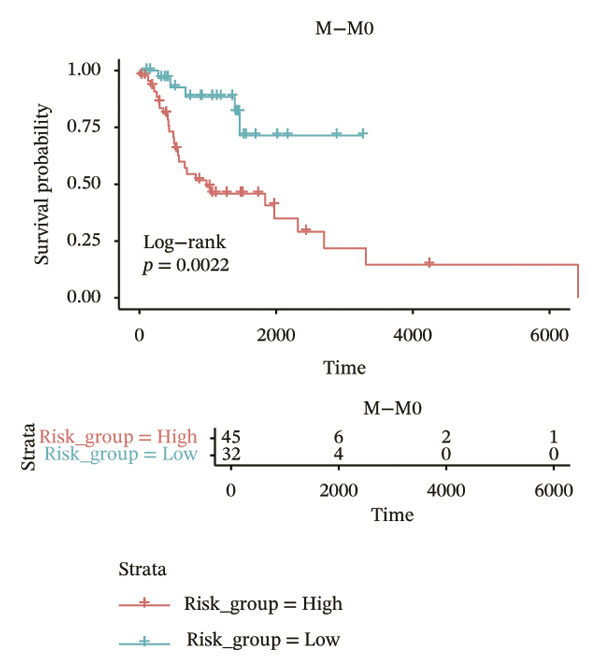
(c)

**Figure:**
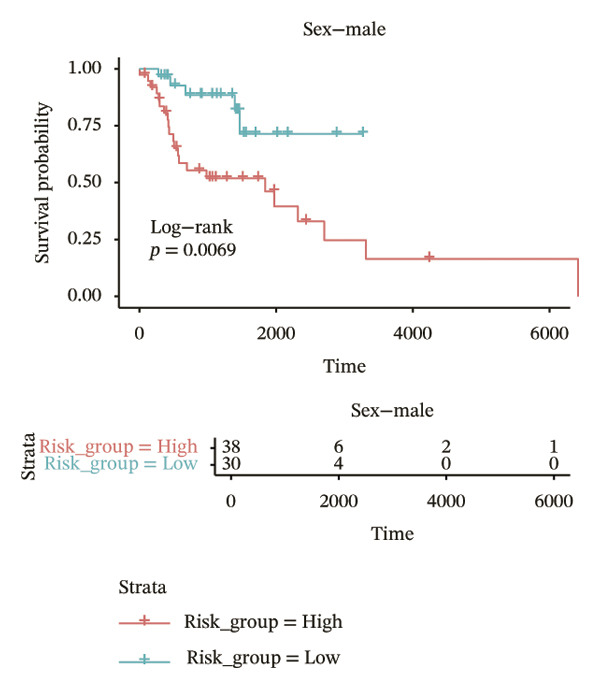
(d)

**Figure:**
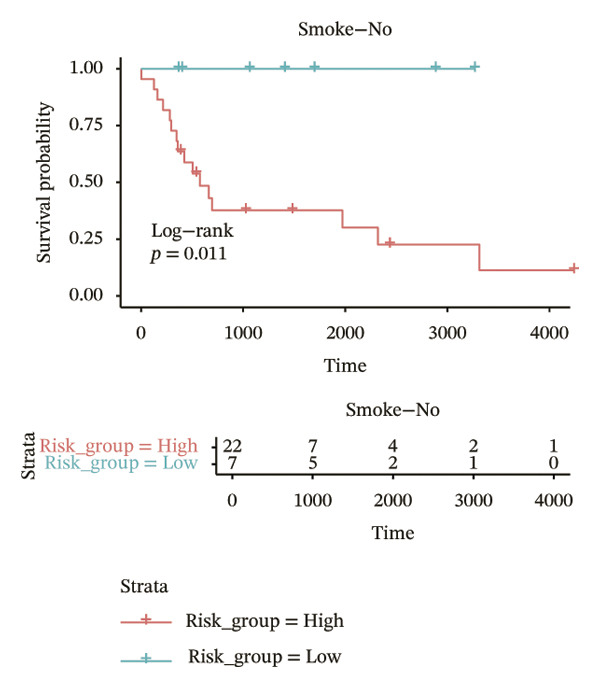
(e)

**Figure:**
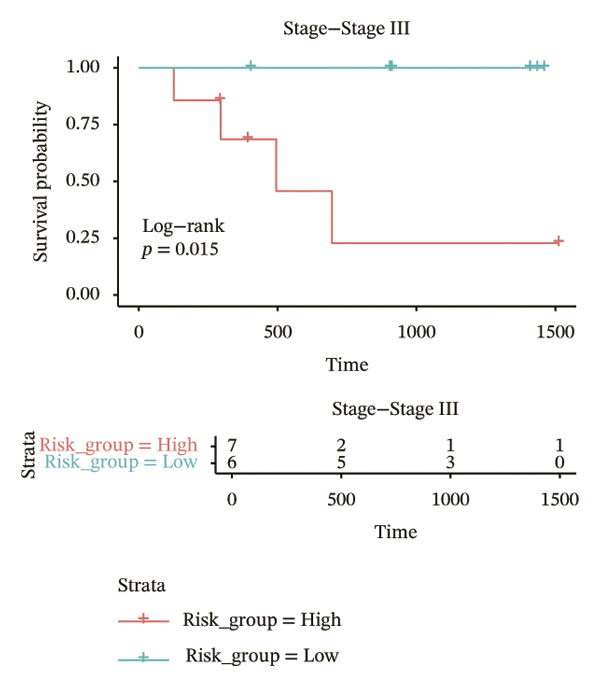
(f)

**Figure:**
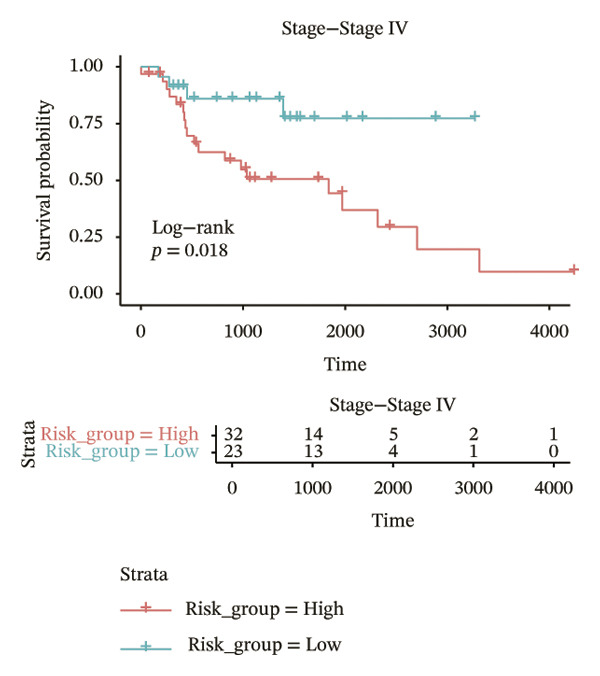
(g)

**Figure:**
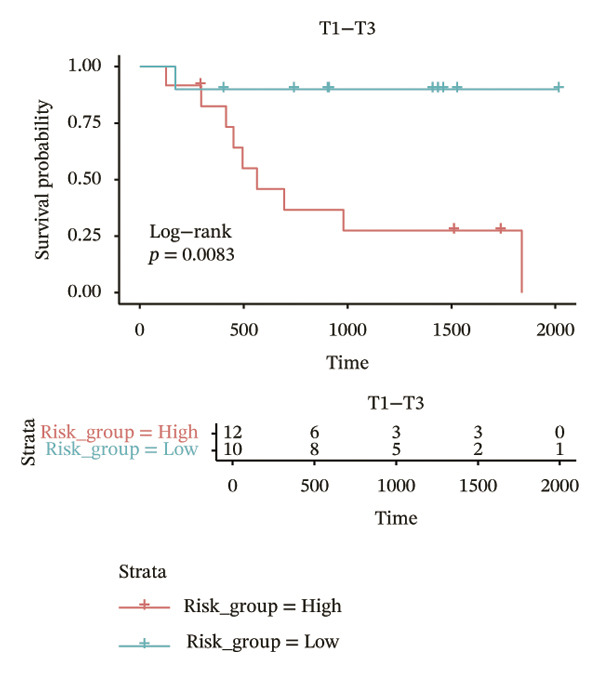
(h)

**Figure:**
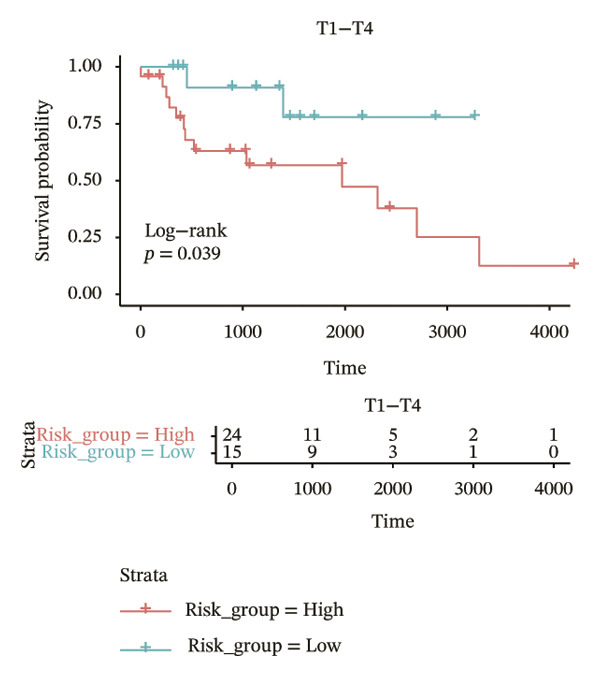
(i)

### 3.5. Enrichment Analysis of Risk Groups

The GSVA result shows that the main functions activated in HRG are spliceosomes, resection and repair, RNA polymerase, and DNA replication, while the main functions activated in LRG are glycosaminoglycan degradation, long‐term depression, glycosaminoglycan biosynthesis of chondroitin sulfate, axon guidance, and vascular smooth muscle contraction (Figure 5(a)) (Supporting Table 6). The GSEA result shows that functions such as axonal guidance and cell adhesion factor CAMs were significantly absent in the HRG (Figure 5(b)) (Supporting Table 7).

FIGURE 5Functional enrichment analysis of the risk model. Results of (a) GSVA and (b) GSEA based on the risk model.

**Figure:**
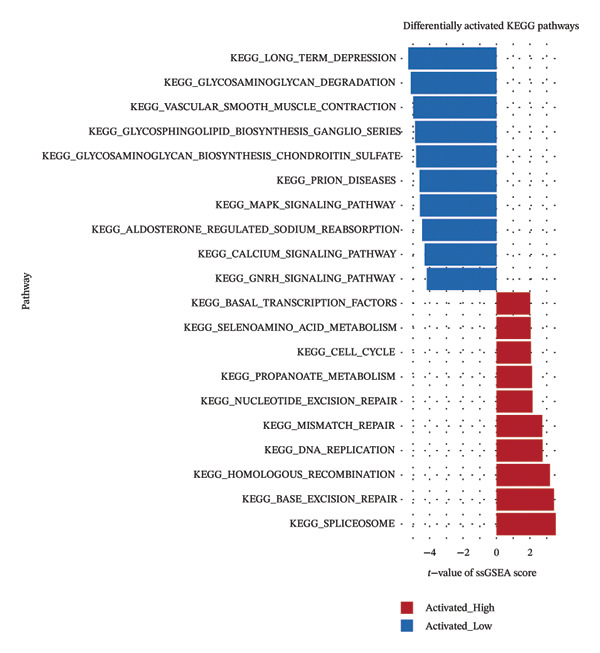
(a)

**Figure:**
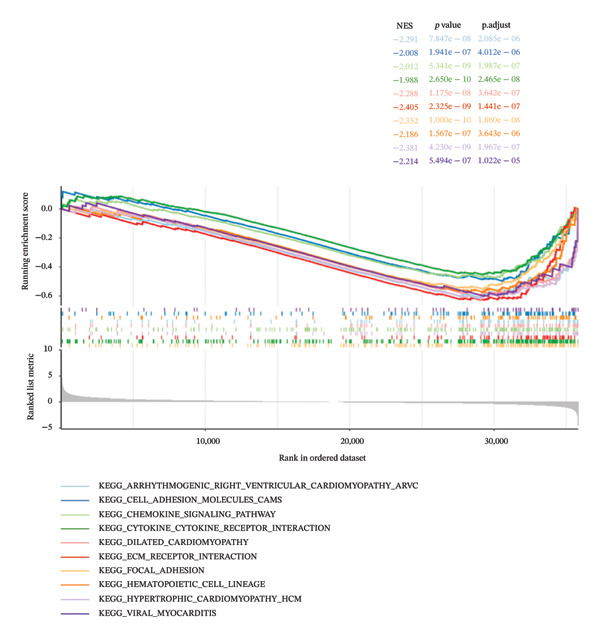
(b)

### 3.6. The Immune System Response of Two Groups

The heatmap presented the enrichment of 22 cell types in two risk groups of LC samples (Figure 6(a)). Only infiltration of T cell follicular helper cells was significantly different (*p* < 0.05). LC development may be influenced by changes in the infiltration profile of these immune cells, with Tfh cells showing higher levels of infiltration in the LRG (adj.*p* = 0.041) (Figure 6(b)). The expression of XRCC3 was positively associated with cell proportion of eosinophils (cor = 0.33, *p* < 0.05) (Figure 6(c)). There were notable discrepancies of stromal (*p* = 0.0076), ESTIMATE (*p* = 0.0126), and immune score (*p* = 0.0433) between HRG and LRG (Figure 6(d)). Additionally, the TIDE scores of the two risk groups also showed significant differences (*p* = 0.023), indicating distinct immune microenvironments and immune escape profiles (Figure 6(e)). The HRG had a higher likelihood of immune escape. Subsequently, we further performed expression difference analysis of immune checkpoints and found that only the expression of TNFRSF25 displayed marked variations between HRG and LRG (*p* < 0.01) (Figure 6(f)). Moreover, its expression was significantly correlated with the expression of CHTF18 (cor = 0.42), FANCG (cor = 0.50), XRCC3 (cor = 0.36), and the risk score (cor = −0.42) (*p* < 0.001) (Figure 6(g)) (Supporting Table 8). This suggested that this immune checkpoint might be a key gene contributing to poor prognosis in HRG.

FIGURE 6Immune infiltration analysis of the risk model. (a, b) Heatmap and bar plot of 22 immune cell types in the HRG and LRG. (c) Correlation between immune cells and key genes. (d) Stromal, immune, and ESTIMATE scores across risk groups. (e) TIDE score comparison. (f) Expression differences of immune checkpoint genes. (g) Correlations of prognostic genes and risk scores with checkpoint expression.

**Figure:**
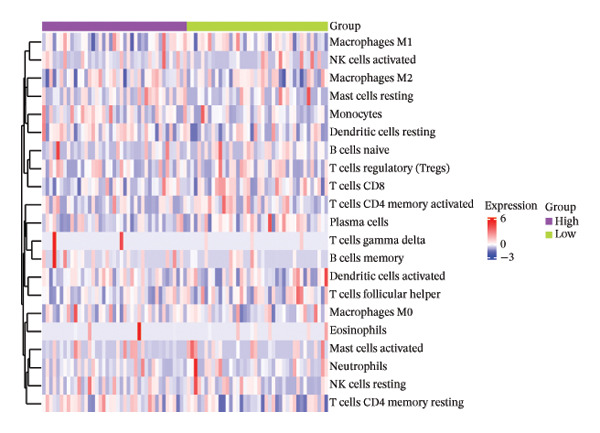
(a)

**Figure:**
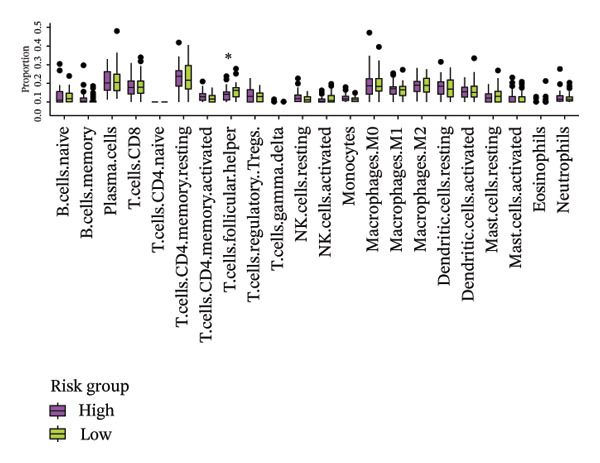
(b)

**Figure:**
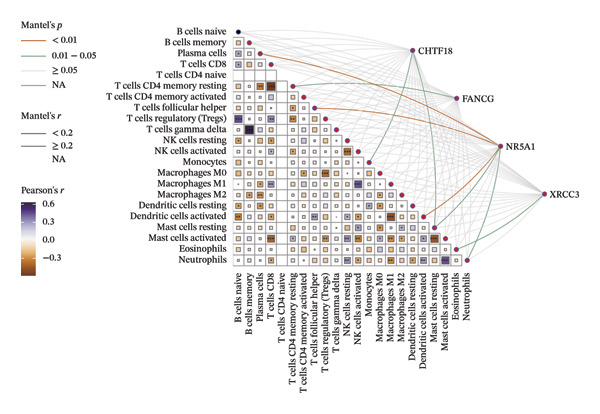
(c)

**Figure:**
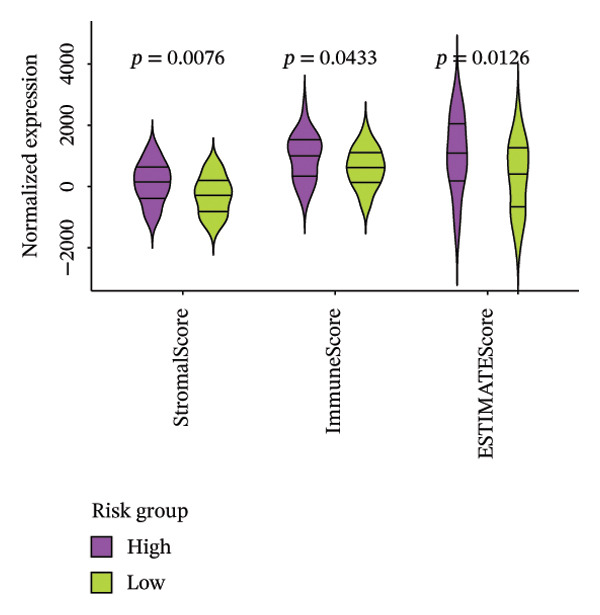
(d)

**Figure:**
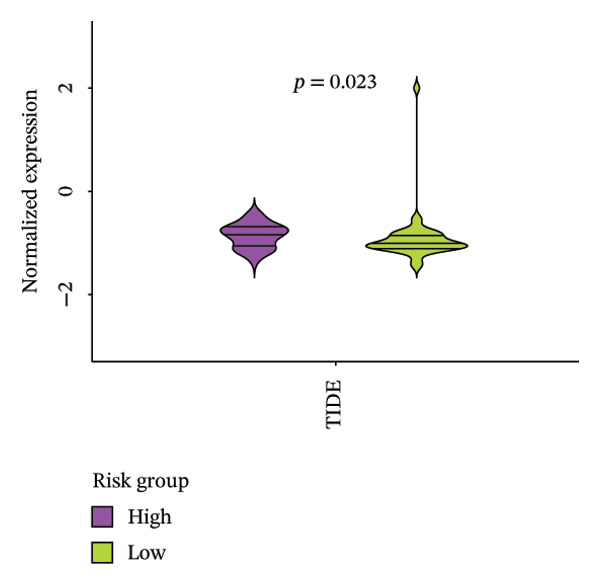
(e)

**Figure:**
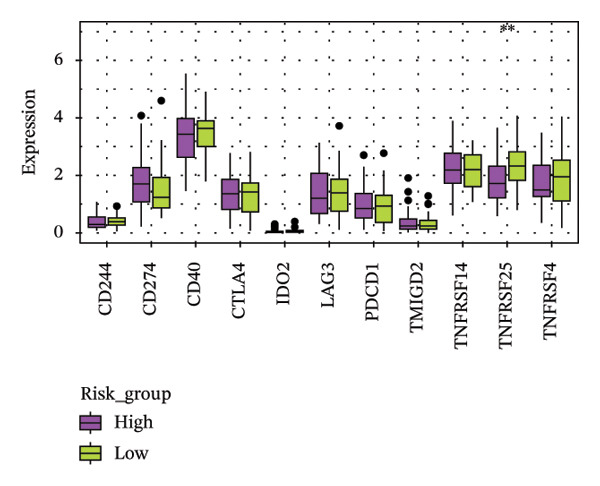
(f)

**Figure:**
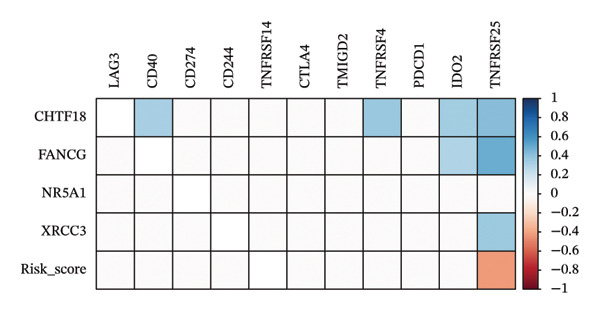
(g)

### 3.7. There Were Differences in Mutation Profiles Between Two Groups

Mutation analysis showed marked variations in TMB scores across the HRG and LRG (*p* < 0.0001). The LRG had higher TMB, indicating that tumor cells were more easily recognized by the immune system (Figure 7(a)). In HRG, 56 samples had mutation data, with 55 samples showing mutations, resulting in a mutation frequency of 98.21%. In the LRG, 53 samples had mutation data, with all 53 samples showing mutations, resulting in a mutation frequency of 100%. In HRG and LRG, TP53 gene mutations accounted for the highest proportion of 80% and 91%, most of which was missense mutations. The second mutated gene in two groups was TTN. The mutation types in HRG were missense mutations, while those in LRG were multiple mutations at the same hit. The third most mutated gene in HRG was KMT2D, while that in LRG was NSD1, and both showed nonsense mutations as the predominant mutation type (Figures 7(b) and 7(c)).

FIGURE 7Gene mutation patterns in the HRG and LRG. (a) Differences in TMB scores between the two groups. (b) Waterfall plot of the top 20 mutated genes in the HRG. (c) Waterfall plot of the top 20 mutated genes in the LRG. In HRG, 56 samples had mutation data, with 55 showing mutations (98.21% frequency). In LRG, 53 samples had mutation data, with all 53 showing mutations (100% frequency).

**Figure:**
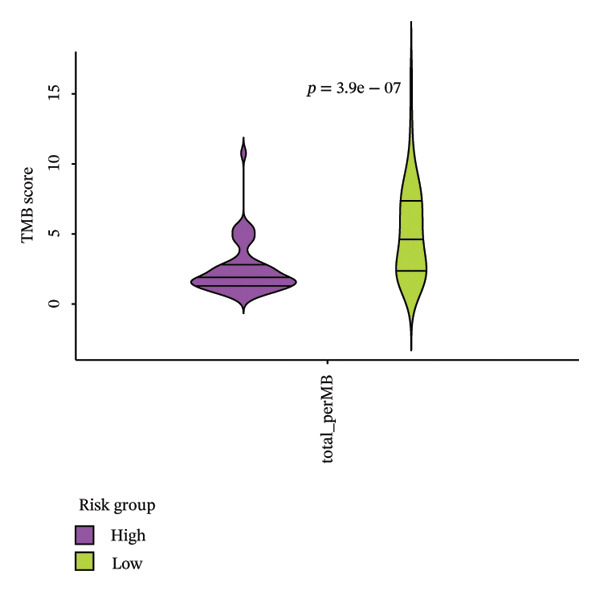
(a)

**Figure:**
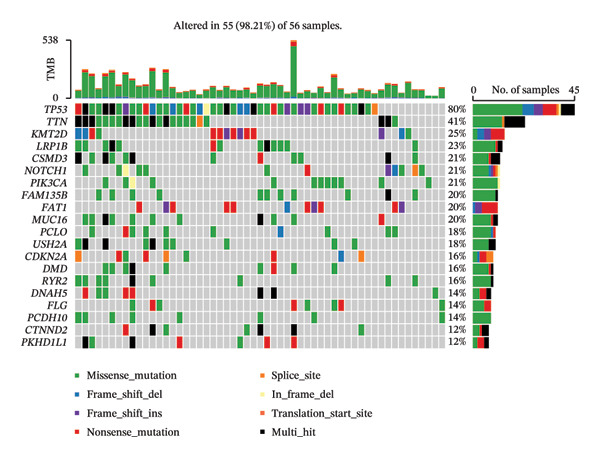
(b)

**Figure:**
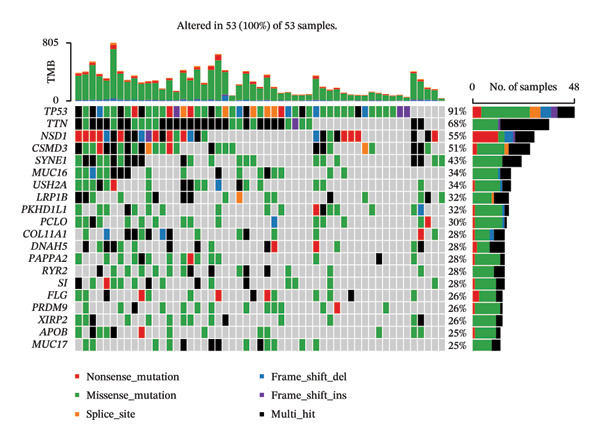
(c)

### 3.8. RT‐qPCR Validation of Prognostic Genes

The RT‐qPCR results from 5 patients, including cancerous and adjacent normal tissues, showed upregulation of three prognostic genes, CHTF18, FANCG, and NR5A1, in the LC group, with *p* values of 0.0131, 0.0309, and 0.0216, respectively. However, XRCC3 expression did not show a significant difference between disease and control groups, with a *p* value of 0.1167. This lack of difference could be due to the limited sample size, which may have reduced the statistical power to detect a significant change (Figures 8(a), 8(b), 8(c), and 8(d)). The clinical results were consistent with most of our bioinformatic analyses, indicating that the bioinformatic analyses were reliable.

FIGURE 8RT‐qPCR validation of prognostic genes. RT‐qPCR results showed that the expression levels of (a) CHTF18, (b) FANCG1, and (c) NR5A1 were significantly upregulated in the disease group, while the upregulation of (d) XRCC3 was not significant.

**Figure:**
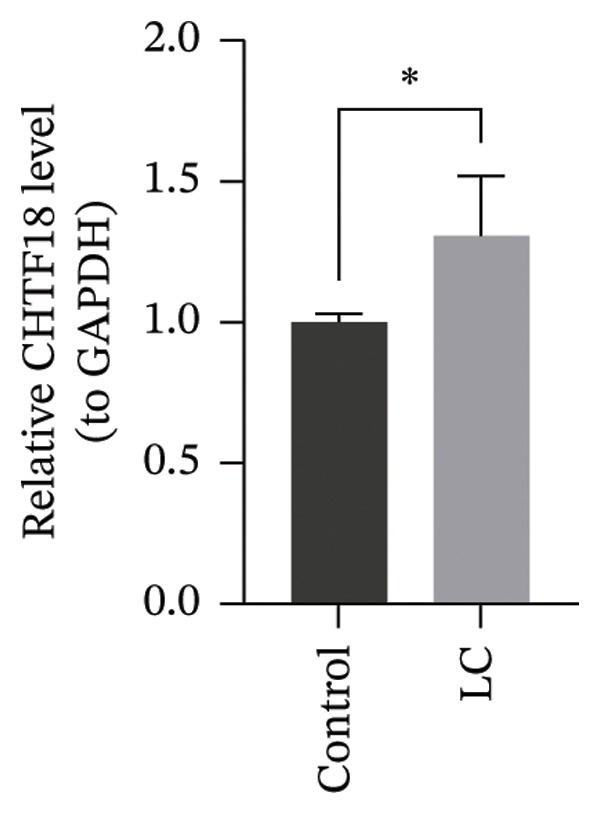
(a)

**Figure:**
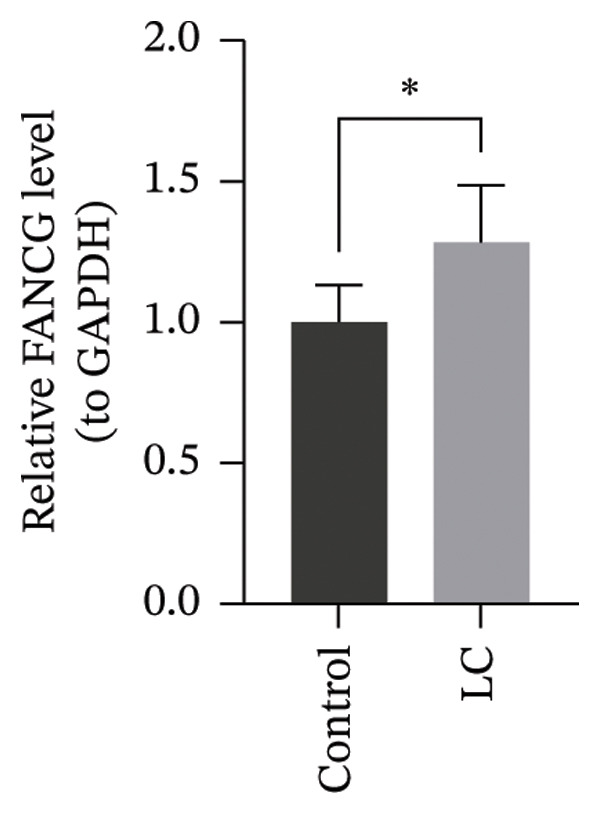
(b)

**Figure:**
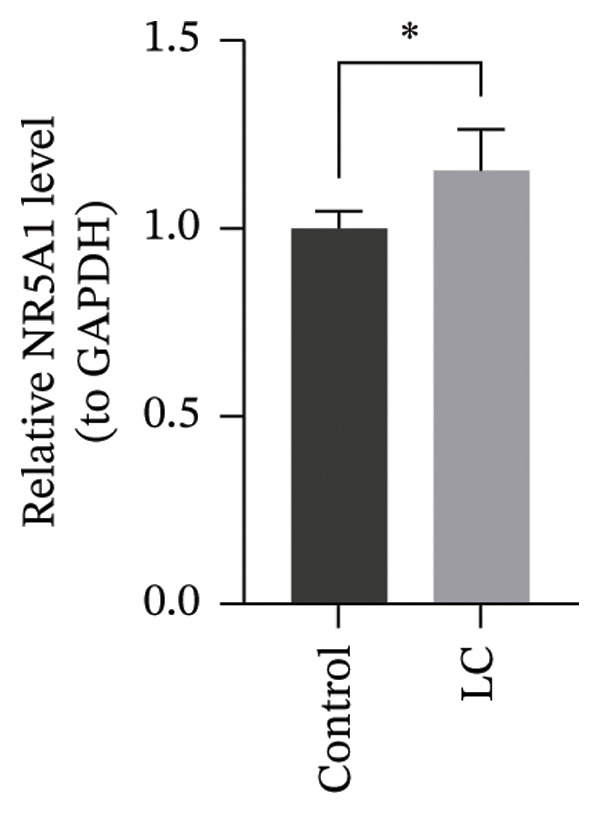
(c)

**Figure:**
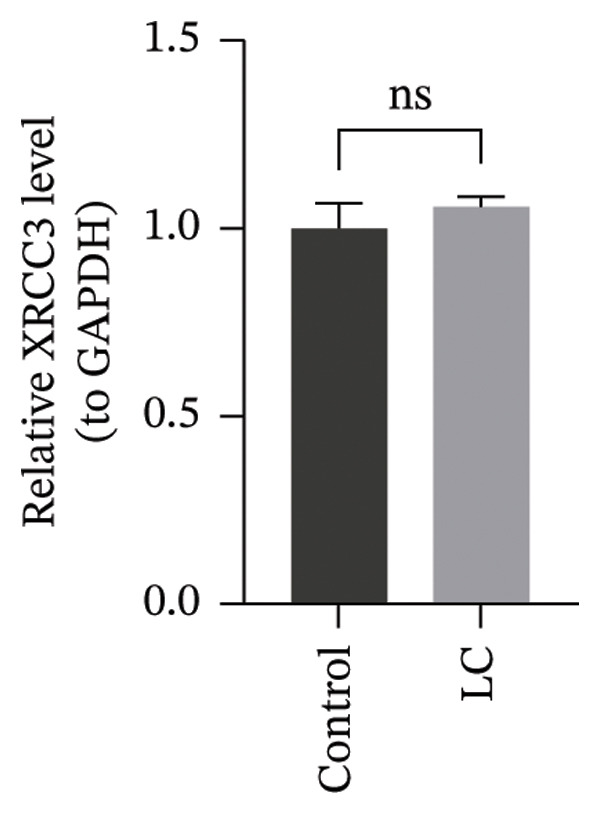
(d)

## 4. Discussion

LC is a malignant tumor originating in the larynx, with a high incidence rate, posing a significant threat to human health [33, 34]. Telomeres are evolutionarily conserved protein–DNA complexes that play a crucial role in regulating the cell division process and ensuring chromosomal stability. Telomerase is highly active in 85%–95% of cancers, enabling cancer cells to proliferate indefinitely and thereby sustaining tumor growth [11, 12, 14, 15, 35]. Despite this, the precise molecular mechanisms linking LC to telomeres remain unclear, highlighting the urgent need for further in‐depth studies to elucidate the role of telomeres in the development and progression of LC. Based on our bioinformatic analysis, we have identified key genes, CHTF18, FANCG, NR5A1, and XRCC3, which may play critical roles in the initiation and progression of LC. While research on these molecules in LC is relatively limited, their potential warrants further investigation.

CHTF18 is a conserved subunit of the replication factor C‐like complex (RLC‐CHTF18). The classical function of RLC‐CHTF18 is to facilitate sister chromatid cohesion and load proliferating cell nuclear antigen (PCNA) onto DNA during DNA replication. It plays a crucial role in chromatid adhesion and DNA replication and is an essential component of DNA replication and repair complexes [36–39]. Although no dedicated studies have examined on the role of CHTF18 in LC, existing research indicates that this gene may be implicated in the development of other cancers, such as endometrial, ovarian, and prostate cancers [40, 41]. This finding suggests that CHTF18 may have significant biological relevance in the development of LC, and further studies are warranted to validate its functional role in this context.

The FANCG gene encodes the FANCG protein, also known as XRCC9, which is a central component of the Fanconi anemia (FA) pathway. This protein plays a crucial role in DNA interstrand crosslink repair and oxidative damage response [39]. During cancer development, mutations in the FANCG gene have been closely associated with various malignancies, including breast cancer [42], ovarian cancer [43], and head and neck cancer [44]. The expression and mutations of FANCG may be closely linked to the mechanisms of cancer initiation and progression, as well as patient survival [45]. These findings suggest that FANCG not only plays a pivotal role in DNA repair but also may be critical to the development and prognosis of LC.

The NR5A1 gene encodes the NR5A1 protein, a transcription factor containing a DNA‐binding domain and a ligand‐binding domain. It regulates transcription by binding to specific response elements in the promoter regions of target genes. NR5A1 plays a crucial role in embryonic development, particularly in the differentiation of the adrenal cortex and gonads, and is a key regulatory factor in steroid hormone synthesis. Additionally, NR5A1 is involved in regulating cell proliferation, apoptosis, and metabolism [46–48]. Research on NR5A1 has predominantly focused on its role in gonadal development, with mutations in this gene being closely linked to disorders of sexual development, gender development abnormalities, and infertility [49–51]. Although studies on the role of NR5A1 in LC are limited, existing research suggests an association between NR5A1 and the development of infantile hemangiomas [52]. These findings indicate that while the precise role of NR5A1 in LC remains unclear, its critical functions in other diseases may provide valuable insights and potential directions for future research into its involvement in LC.

The XRCC3 gene encodes the XRCC3 protein, which belongs to the RAD51 family and is involved in repairing DNA double‐strand breaks. As a critical homologous recombination factor, XRCC3 plays a vital role in the repair of DSBs and in processing stalled replication forks. XRCC3 has been identified as an essential tumor suppressor in cellular processes [53]. Existing studies have shown that polymorphisms in the XRCC3 gene are closely associated with the development of gastric cancer, with a particularly significant correlation observed in Asian populations [54]. Additionally, polymorphisms in this gene have also been linked to increased cancer risk in ovarian cancer, specifically in Caucasian populations [55]. These findings underscore the critical role of XRCC3 in cancer development, particularly in maintaining DNA repair and genomic stability, which is of considerable importance for understanding its potential role in LC. Taken together, these observations highlight potential roles of these genes in LC tumorigenesis and progression, warranting further experimental validation.

The HRG appears to be enriched in pathways such as the spliceosome, calcium signaling, DNA replication, and nucleotide excision repair, which may suggest their potential involvement in HRG tumorigenesis. In contrast, the LRG shows enrichment in the glycosaminoglycan biosynthesis–chondroitin sulfate and endocytosis pathways, indicating a potentially distinct molecular profile. The involvement of the spliceosome pathway suggests that posttranscriptional regulation might play a role in HRG, potentially contributing to the complexity of the cellular proteome and tumor development. Particularly in cancer, RNA splicing is a highly intricate and finely regulated process, prone to alterations during tumorigenesis [56, 57]. Oncogenes and tumor suppressors can modulate posttranscriptional mechanisms, thereby promoting tumor cell survival. In the HRG of LC patients, aberrations in the spliceosome may disrupt the transcriptional process, thereby fostering cancer cell proliferation or overexpression of proto‐oncogenes, which in turn leads to poor prognosis for the patients.

The calcium signaling pathway plays a crucial role in cell proliferation, differentiation, apoptosis, and metabolism [58]. Telomere shortening is closely associated with the cell cycle and apoptosis. Studies have shown that calcium ions are integral to the regulation of telomerase activity [59, 60]. The enrichment of this pathway in the HRG suggests that intracellular calcium signaling is more actively regulated, a phenomenon consistent with previous research and further supporting the notion of a close relationship between calcium signaling and telomere function. Furthermore, the HRG is also enriched in pathways related to DNA replication and repair. DNA replication and repair are core functions associated with telomeres [61]. The enrichment of the HRG in pathways such as DNA replication and nucleotide excision repair suggests enhanced DNA synthesis and damage repair capabilities. This indicates that telomeres play a crucial role in the unchecked proliferation of tumor cells, thus supporting the validity of our risk model based on T‐RGs.

The enrichment of the glycosaminoglycan biosynthesis–chondroitin sulfate pathway in the LRG suggests a stronger capacity for extracellular matrix synthesis and repair. Glycosaminoglycans are key components of the extracellular matrix, playing a pivotal role in tissue repair and intercellular interactions. The enrichment of this pathway may be associated with the cells’ adaptation to the tumor microenvironment [62], indicating that the LRG exhibits greater stability of the extracellular matrix and enhanced tissue recovery capabilities. Endocytosis, a crucial mechanism for cellular uptake of external substances, also plays a significant role in signal transduction and material transport and is closely linked to tumor initiation and progression [63]. The enrichment of this pathway in the LRG reflects the potential regulation of nutrient uptake and signal transduction, which helps maintain cellular stability and a relatively suppressed growth state within the tumor microenvironment, thereby decelerating tumor progression. The enrichment of these pathways further validates the accuracy and biological significance of the prognostic risk model based on T‐RGs in LC.

In our immune infiltration analysis, we observed a significant difference in the infiltration of follicular helper T cells (Tfh) between the HRG and LRG, which allows us to further explore the role of these cells in immune responses. Tfh cells regulate the germinal center response, influencing the generation of high‐affinity antibodies, memory B cells, and long‐lived plasma cells derived from the germinal center. These processes form the foundation of long‐lasting humoral immunity [64, 65]. In this study, we found that Tfh cell infiltration was significantly higher in the LRG, indicating a more effective humoral immune response in this group. This finding aligns with the favorable prognosis observed in the LRG. These results suggest that Tfh cells may play a crucial role in the immune microenvironment of LC, and their degree of infiltration may be closely associated with patient prognosis. Therefore, Tfh cells not only play a key regulatory role in immune responses but also may serve as potential biomarkers for assessing prognosis and immunotherapy response in LC patients. This provides a new direction for future research, particularly in the context of personalized treatment and the application of immunotherapy.

In this study, four relevant prognostic genes (CHTF18, FANCG, NR5A1, and XRCC3) were identified using bioinformatic approaches, and a prognostic model was constructed. It explores the potential associations of these genes in LC prognosis, aiming to provide new directions for the treatment and drug development of LC. However, we must acknowledge certain limitations in this study. First, the absence of external validation is a significant limitation. The analysis primarily relied on the TCGA database, and since no other datasets with survival information were identified, the findings may be influenced by dataset‐specific biases or spurious associations. Additionally, the small sample size used in the RT‐qPCR validation limits the robustness and generalizability of these results. The reliance on a single public dataset (TCGA‐HNSC) introduces the risk of potential subsite heterogeneity, as this dataset covers a broad range of head and neck cancers, which may not fully represent the specific characteristics of LC. Moreover, the lack of functional validation of the identified prognostic genes further restricts our ability to confirm their mechanistic roles in LC. This absence of experimental validation and cross‐validation with external datasets, such as GEO or clinical sample data from different ethnicities or populations, weakens the reliability and clinical translational potential of the findings. To further strengthen the findings of this study, future research should include several key directions. First, multicenter validation is necessary to ensure the generalizability of the results across diverse populations and clinical settings. Additionally, mechanistic experiments are essential to provide a deeper understanding of the biological roles of the identified prognostic genes, thereby confirming their relevance in LC pathogenesis. Furthermore, assessing telomere length alongside gene expression would offer valuable insights into the relationship between telomere biology and gene regulation in cancer, enhancing the comprehensiveness of the study. These future directions will not only validate our current findings but also contribute to the development of more effective diagnostic and therapeutic strategies for LC.

**Nomenclature**
TCGA: The Cancer Genome Atlas
DEGs: Differentially expressed genes
OS: Overall survival
PPI: Protein–protein interaction
LC: Laryngeal cancer
T‐RGs: Telomere‐related genes
GO: Gene Ontology
KEGG: Kyoto Encyclopedia of Genes and Genomes
TMB: Tumor mutation burden
HRG: High‐risk group
LRG: Low‐risk group
GSEA: Gene set enrichment analysis
GSVA: Gene set variation analysis
BP: Biological process
CC: Cellular component
MF: Molecular function

## Author Contributions

Yesong Cheng conceived and designed the study. Xiaolong Tang and Lin He collected the clinical and transcriptomic data. Yesong Cheng, Xiaolong Tang, and Dingqiang Huang performed the bioinformatic analyses and interpreted the results. Feipeng Zhao and Xiangyang Shi carried out the laboratory experiments and validated gene expression by RT‐qPCR. Wensong Tang contributed to data visualization and figure preparation. Yi Liu assisted in statistical analysis and critical revision of the manuscript. Wujun Zou drafted the manuscript and coordinated revisions. Yi He provided funding for the study and contributed to the critical revision of the manuscript.

## Funding

This work was supported by the Research Development Fund Project of North Sichuan Medical College (No. CBY22‐QNA12), Science and Technology Development Plan of the Affiliated Hospital of North Sichuan Medical College (No. 2023PTZK008), Nanchong City Science and Technology Project, Special Project for University‐Enterprise Science and Technology Strategic Cooperation (No. 22SXQT0077), Scientific Research Development Plan Project of the Affiliated Hospital of North Sichuan Medical College (No. 2024GC004), Guang’an City Science and Technology Innovation Project (No. 2024SYF17), Research and Development Project of Ganzi County People’s Hospital (No. 2025GZ001), and Sichuan Provincial Medical Research Youth Innovation Project (No. 2025_QNKY_0769).

## Disclosure

All authors have read and approved the final version of the manuscript.

## Ethics Statement

This study was reviewed and approved by the Medical Ethics Committee of Chengdu Second People’s Hospital (Approval No. [KY]PJ2024144). The requirement for written informed consent was waived by the committee, as the study involved only the use of archived tissue samples and posed no additional risk to participants.

## Conflicts of Interest

The authors declare no conflicts of interest.

## Supporting Information

Additional supporting information can be found online in the Supporting Information section.

## Supporting information


**Supporting Information 1** Supporting Table 1: Summary of T‐RGs sourced from TelNet (TelNet score ≥ 1, v2024.11.17).

**Supporting Information 2** Supporting Table 2: Patient’s basic information.

**Supporting Information 3** Supporting Table 3: Clinical features of LC patients from the TCGA database.

**Supporting Information 4** Supporting Table 4: Differentially expressed genes (DEGs) identified between LC and control samples in the TCGA‐HNSC training set.

**Supporting Information 5** Supporting Table 5: Summary of GO and KEGG enrichment terms for differentially expressed genes.

**Supporting Information 6** Supporting Table 6: GSVA results showing the main pathways and functions activated in HRG and LRG groups.

**Supporting Information 7** Supporting Table 7: GSEA analysis results based on the risk model.

**Supporting Information 8** Supporting Table 8: Correlation analysis of CHTF18, FANCG, NR5A1, XRCC3, and the risk score with immune checkpoints.

## Data Availability

The datasets analyzed in this study are publicly available in the Cancer Genome Atlas (TCGA) database (https://portal.gdc.cancer.gov/).
